# 

**Published:** 1878-10-20

**Authors:** 


☞All communications relating to the business of The Record for the years 1877 and 1878, must be addressed DR. R. C. WORD, Managing Editor Southern Medical Record, Atlanta, Ga.

☞Brief and practical communications are solicited on all subjects pertaining to medicine; also reports of cases in practice.

☞Send money by check, postal order or registered letter.

☞Write your name, post-office, county and State plainly.

RECEIPTED.—Drs. J B. Smith 6 months, J. T. Sego, E. A. Anderson, H. S. Sutberland, R. H. Coleman, W. S. Sims, J. W. Heacock, J. W. Price, John Coston, H, M. Talley. J. N. Siddons, L. B. Creath, S. M. Wood, J. H, Calfee, H. T. Shiell, T. T. Key.

### NOTICE.

Parties in arears will please remit their subscriptions.

